# Natural history of glaucoma

**DOI:** 10.4103/0301-4738.73682

**Published:** 2011-01

**Authors:** Ying Pan, Rohit Varma

**Affiliations:** Doheny Eye Institute, University of Southern California, Los Angeles, CA, USA

**Keywords:** Angle closure glaucoma, natural history definition of glaucoma, open angle glaucoma

## Abstract

**Purpose:**

To present an overview of the recent observations and research that shed light on the understanding of open and closed angle glaucoma.

**Methods:**

Literature review.

**Results:**

Glaucoma is a major eye problem afflicting millions of people worldwide. As the population increases, the number of people with glaucoma also increases, with glaucoma becoming an increasing public health concern. This paper presents the natural history of open angle and angle closure glaucoma. We examine the glaucomatous progression in terms of changes in optic disk morphology and visual fi elds as well as the risk factors for progression.

**Conclusions:**

This present review highlights the magnitude of glaucoma globally and the need for a greater understanding of this disease and its natural progression.

Open and closed angle glaucoma are leading causes of blindness. With aging of the population, the number of people with glaucoma is expected to rise, posing a substantial public health challenge worldwide. Understanding the natural history of glaucoma is essential to our clinical practices

The manifestations of glaucoma range from mechanical angle closure of outflow structures in patients with angle closure glaucoma (ACG), who typically present with ocular pain and acute visual loss, to increased resistance of outflow in patients with open angle glaucoma (OAG), who are often asymptomatic. Although glaucoma embodies a diverse group of diseases, all these diseases share common characteristics, the hallmarks of which include progressive irreversible damage to the optic nerve head and the retinal ganglion cells with corresponding visual field loss.

Primary OAG is defined as a chronic optic neuropathy with characteristic changes in the optic disc and visual field. Risk factors for OAG include older age, black race, family history (first-degree relative), thinner central corneal thickness, myopia and elevated intraocular ocular pressure (IOP). ACG is characterized by the opposition of the iris to the trabecular meshwork, resulting in blockage of the aqueous outflow. Risk factors for developing angle closure include Asian race, female gender and advanced age. Anatomic features predisposing to angle closure are hyperopia, anterior iris insertion and shallow anterior chamber.

It is important to note that the definition of glaucoma has evolved from a disease of eye pressure to a disease of optic neuropathy. An elevated IOP in the affected eye is now seen as a risk factor for glaucoma rather than its cause. Presently, however, IOP is the only modifiable risk factor that can be used to prevent progressive optic neuropathy.

## Magnitude of Glaucoma Worldwide

Glaucoma is the leading cause of irreversible blindness worldwide, and the second most common cause of blindness after cataracts.[1,2] It is responsible for 14% of blindness worldwide.[3] It afflicts almost 70 million people, of whom 10% are believed to be bilaterally blind.[2]

Several population-based studies have contributed to our understanding of the incidence and prevalence of OAG within defined populations in the United States and other countries. In the Baltimore Eye Survey, the prevalence of OAG was significantly higher in blacks (4.7%) than in whites (1.3%).[4] The Los Angeles Latino Eye Study[5] found that Latinos in the United States have a prevalence of OAG of 4.7%. The prevalence of OAG in Asians varies widely, perhaps in part because the term Asian encompasses broad racial and ethnic categories. Rudnicka *et al*. documented OAG rates in Asia to range from 1 to 4%,[6] whereas Ramakrishnan *et al*. found the prevalence of OAG in India to be 1.7%.[7]

In Asian populations, ACG is the main cause of morbidity from glaucoma. ACG blinds 10-times more people than OAG does, and the worldwide incidence of ACG is growing.[8] While ACG represents only 10–15% of all glaucomas in the black and white populations, it accounts for a significant percentage of glaucomas that occur in Asian populations. The rate of ACG among Chinese is three-times that of OAG.[1] Approximately 91% of bilateral blindness in China is due to ACG.[9] Vijaya *et al*. found that 2.75% of the population had angle closure and 0.88% had ACG.[10] The Andhra Pradesh Eye Disease Survey in south India suggests that 0.7% of the population over 30 years of age has ACG.[11]

## Glaucomatous Morphology of the Optic Nerve

Glaucoma damages the ganglion cell and its respective axons, which comprise the retinal nerve fiber layer (rNFL). This results in progressive and asymmetric changes in the optic cup, with corresponding visual field loss. Typically, structural changes occur before functional loss. Up to 40% of the retinal nerve fibers may be destroyed before detectable changes in visual field. The morphology of these rNFL defects follows the normal structural pattern of the rNFL in the retina. Normally, rNFL has a striated appearance, radiating from the optic disk, and is thickest in the superior and inferior poles, compared with the nasal and the temporal poles. Glaucomatous rNFL changes can present as focal wedge-shaped defects of varying width radiating from the optic nerve head or as diffuse loss of the striations in rNFL.[12] Because glaucoma tends to afflict the superior and inferior nerve fibers preferentially, focal loss is often detected in these areas.

Disc changes present with a variety of characteristic patterns. As ganglion cells and their axons are destroyed, the neural rim begins to thin. Typically, localized thinning in early glaucoma can lead to focal atrophy of the neural rim, known as focal notching. This tends to occur in the inferotemporal region of the optic nerve head because of preferential loss of the inferior nerve fibers. This is followed, to a lesser extent, by focal neural loss and atrophy in the superotemporal region. As a result, the optic cup usually enlarges in a vertical or oblique fashion. As the glaucomatous process progresses, the temporal rim becomes involved. The nasal quadrant is the last to be affected. Early glaucomatous damage can also lead to progressive, generalized and concentric expansion of the nerve cup. In some cases, early glaucomatous optic atrophy presents with deepening of the cup, exposing the underlying lamina cribrosa. In other cases, early glaucoma can be evidenced by saucerization of the disc, in which shallow sloping and cupping extend to the margins of the disk. Progressive glaucoma results in axonal loss and backward bowing of the lamina cribrosa, leading to enlargement and/or excavation of the cup. Loss of all neural rim tissue with exposure of the laminar pores can be seen in advanced glaucoma. Complete cupping with undermining of the neural rim produces a bean pot appearance, with a pale disc and vessels that bend at the margins of the disc.

Vascular signs of glaucomatous optic atrophy include splinter hemorrhages that result due to loss of axons at the optic nerve head and reflect progressive rNFL damage. They occur more commonly in patients with normal-tension glaucoma (NTG) than those with primary OAG, with a cumulative incidence of 35.3% and 10.3%, respectively.[13] The most common location for these hemorrhages is the temporal rim, followed by the inferior and superior rim. Rarely, splinter hemorrhages occur nasally. They are most often seen in the early to middle stages of glaucoma and are a prognostic sign of progressive disease. The hemorrhages leave behind a focal area of rNFL defect, focal notching and a corresponding visual field defect.[14,15] The disappearance of neural rim can lead to overpassing vessels. The bending of the retinal vessels along the edge of a disappearing rim is termed bayoneting. Circumlinear vessels may be also bared from the margin of the cup. In advanced glaucoma, the central vessels can be nasally displaced.

## Glaucomatous Visual Field Progression

Early glaucoma can create mild, diffuse depression in the visual fields and/or localized visual field defects. In these earlier stages, peripheral changes in visual fields may be the only detectable abnormality. Increasing scatter and fluctuation is often noted. Isolated defects tend to occur in the superior half of the visual field because of the susceptibility of the inferior poles of the optic nerve in early glaucomatous damage. Although central vision is preserved during the early course of glaucoma, defects can involve the fixation point. Isolated paracentral defects can appear as the initial glaucoma defect in 41% of the patients.[16]

Progression in visual fields can occur in a variety of ways. There can be gradual but steady decrease in retinal sensitivity affecting the field uniformly. Initial defects that were shallow can coalesce, extend, deepen and enlarge into nasal steps, arcuate scotomas or complete altitudinal defect. New defects can also appear with further progression. For example, in advanced glaucoma, arcuate scotomas can manifest superiorly and inferiorly, forming a double arcuate scotoma. This double arcuate scotoma comes together nasally at the horizontal meridian, creating the central and temporal islands seen in advanced glaucoma. With the destruction of the remaining areas of the macular fibers and the nasal retina, these islands continue to disappear until they are extinguished. Temporal islands may be more resistant and may persist after central islands are lost. However, these too can be destroyed, leaving patients with complete visual loss.

## Natural History of Open Angle Glaucoma

The lack of symptoms in OAG plays a large role in delaying its detection and diagnosis. Typically, OAG is slowly progressive, remaining asymptomatic until late. By the time OAG becomes symptomatic, severe and irreversible damage has usually occurred to the visual field in one or both eyes. The rate of progression of the visual field defect varies in patients, and treatment of the glaucoma may not completely halt the visual field loss.[17] Some patients progress despite aggressive therapy.[18]

The incidence of blindness 20 years after the initial diagnosis of OAG has been estimated at 27% for one eye and 9% for both eyes in a primarily white population.[19] Data from population-based, cross-sectional studies revealed that for patients with OAG, the mean change in visual field testing for European-derived, Hispanic, African-derived and Chinese was –1.12, –1.26, –1.33 and –1.56 dB/year, respectively. The differences in the mean deviation (MD) were not statistically significant by ethnicity. Because some participants were treated, the data cannot be used to represent the natural history of OAG.[20]

The Ocular Hypertension Treatment Study elucidated the natural history of OAG by identifying the rate of conversion from ocular hypertension to primary OAG and the impact of treating IOP (decreasing IOP more than 20% from baseline) on the development of OAG. After 60 months of follow-up, conversion to OAG from ocular hypertension was 4.4% in the treated group as compared with 9.5% in the untreated group. Thus, a protective effect of 54% was seen with treatment. However, over 90% of the untreated subjects did not develop visual field or disc changes consistent with OAG during this time. To prevent one patient with ocular hypertension from developing glaucoma, 19 would need to be treated unnecessarily if the risk factors were ignored. Baseline factors associated with the conversion to OAG included advanced age, elevated IOP, central corneal thickness thinner than the study mean, increased cup-to-disc ratio and increased pattern standard deviation on the visual field.[21,22]

Data from individuals in the Early Manifest Glaucoma Trial (EMGT) randomized to the no-treatment group shed light on the natural course of newly detected OAG and can be used to predict the likelihood of visual loss from glaucoma. After 4 years of follow-up, 49% of the individuals without treatment progressed, compared with 30% with treatment (an average IOP lowering of 25%).[23,24] After 6 years of follow-up, 68% of the untreated patients showed definite visual field progression, with an overall median time to progression of 42.8 months. The study also revealed a very large variation in time to progression among the subjects. Some progress rapidly, with a deterioration in the MD index of greater than 10 dB per year; others did not progress at all, even after lengthy follow-up. Of those individuals in the high-tension glaucoma (HTG) group (with elevated IOP ≥21 mm Hg), 74% had progressed, with a median time to progression of 44.8 months, while 56% of those individuals with NTG progressed, with a median time to progression of 61.1 months. Of the pseudoexfoliation patients (PXEG), 93% progressed, with a median time to progression of 19.5 months. Thus, the visual field loss progressed for most of these patients; the majority progressed slowly, but a minority progressed rapidly. Specifically, in the PXEG group, the MD on automated visual field testing was –3.13 dB/year. The perimetric MD for the NTG group and the HTG group was –0.36 dB/year and –1.31 dB/year, respectively.[25] Large variations existed between the rates of progression in visual field for HTG, NTG and PXEG as well as among subjects within each group.

This variability in clinical course was also found by the Collaborative Normal Tension Glaucoma Study (CNTGS). Similar to the EMGT, the CNTGS documented the natural course of untreated NTG.[26] The study specifically focused on patients with glaucomatous optic nerve damage and visual field loss accompanied by IOP in the normal range. While some believe that NTG represents a distinct variety of glaucoma from primary OAG, the two most likely represent a continuum of glaucomas. After 5–7 years of follow-up, progression of the visual field defect was noted in 60% of those individuals with untreated glaucoma with optic nerve damage, visual loss and IOP under 21 mmHg. Treatment targeting IOP lowering of >30% decreased the progression rate to 20%.[26] Most cases progressed slowly, requiring several years to demonstrate progression; in other cases, deterioration manifested within 1 year. The mean estimated slope of the MD index deterioration for all untreated subjects was –0.41 dB/year. However, the MD index ranged from –0.2 dB/year to –2 dB or more/year. This 10-fold range reflects the broad range in the rates of deterioration.

Because the course of glaucomatous progression is highly variable, identifying factors that predict progression can help guide clinical practice and patient treatment and monitoring. In the EMGT, faster and greater progression was noted in older patients (≥68 years of age) when compared with younger patients. Frequent disc hemorrhages predict faster progression, as did bilateral disease and greater visual field loss at initial diagnosis, as measured by perimetric MD. PXEG glaucoma, when compared with NTG and HTG, was also noted to be a more aggressive disease, with a mean progression rate corresponding to full-field blindness within 10 years. In addition, glaucoma patients with higher IOP are more likely to progress rapidly than those with IOP <21. NTG patients progressed more slowly and had a lower risk of rapid evolution to blindness. Therefore, the immediacy and aggressiveness of therapy for these patients may be less than that for patients with HTG and PXEG. That being said, high intragroup variability exists and, therefore, treatment should be guided by individual presentation.

The EMGT and the CNTG are the only two prospective studies that studied large groups of people with glaucoma without treatment. These two studies have provided important data on the natural course of OAG and on its risk factors for progression. Patients need to be monitored carefully after being diagnosed with glaucoma to determine the rapidity of glaucoma progression. Individualized treatment plans must be tailored to patients and to their rate of progression.

## Natural History of Angle Closure Glaucoma

Although OAG is more common worldwide, ACG causes more serious loss of vision than OAG.[9] ACGs are characterized by apposition of the peripheral iris against the trabecular meshwork, resulting in obstruction of the aqueous outflow. The main mechanisms of closure are pupillary block, plateau iris, lens-related and retrolenticular causes. The most common cause is pupillary block.

ACG may be divided into acute, subacute and chronic ACG. Although they represent different clinical manifestations, they can occur at different times in the same person. In acute ACG, closure of the angle occurs suddenly, resulting in rapid rise in IOP. The affected person may present with dramatic symptoms of severe ocular pain, nausea, vomiting, headache and blurred vision. Subacute or intermittent ACG occurs when episodes of pupillary block resolve spontaneously and can recur repeatedly over time. Chronic ACG develops when the angle narrows slowly and results in scarring between the peripheral iris and the trabecular meshwork.

The natural history of ACG has been subdivided into three stages: (1) an anatomically narrow angle without elevated IOP, abnormal visual fields or peripheral anterior synechia (primary angle closure suspect [PAC]), (2) development of peripheral anterior synechia or a closed angle with elevated IOP, labeled PAC and (3) development of an anatomical angle closure with glaucomatous optic nerve and visual field changes, termed primary angle closure glaucoma (PACG).[27]

Although the prevalence and pattern of disease varies across different parts of the world, the majority of those with ACG will be Asian due to their anatomical predisposition. Data on the natural history of ACG are limited. Large population-based data on the disease progression are nonexistent. In one small study, 22% of the normal patients with narrow angles developed synechial angle closure (64%) or appositional angle closure (36%) over a period of 5 years.[28] Of 28 subjects who were identified as having PAC, eight progressed to PACG within 5 years. Only one of the nine participants who underwent laser peripheral iridotomy (LPI) progressed compared with seven of 19 subjects who refused the laser iridotomy.[28] Publications on response to treatment provide important insight into the natural history of angle closure. The first-line treatment of LPI relieves the relative pupillary block element. The response to LPI and the long-term course of PACG appears to vary by race. Studies found that LPI in Caucasian subjects with ACG were more likely to effectively prevent the subsequent need for surgical intervention than LPI in Asian subjects. Intraocular pressure was controlled with LPI alone in 65–76% of eyes, with only 0–13% of the eyes requiring subsequent filtering surgery.[29–33] In Asian populations, however, Alsagoff *et al*. found that the majority of eyes with established ACG required antiglaucoma medications or filtering surgery, despite undergoing treatment with LPI.[34] The disease in Asians appears to be more aggressive. Even after laser iridotomy for eyes with narrow angles, the rates of progression to ACG can be significant. A decade after treatment for acute PAC, 47.8% of the patients developed glaucomatous optic neuropathy.[35] Aung *et al*. found that several years after the initial attack of acute angle closure in Asian subjects, 17.8% were blind in the affected eye and half had blindness caused by the advanced glaucoma.[35] Thus, Asian patients are at a higher risk of further glaucomatous damage even after patent LPI and would benefit from long-term follow-up.

## Glaucoma Screening and Prevention

As the number of people with glaucoma is expected to grow, glaucoma will become an increasing public health problem in the coming decade. Undiagnosed glaucoma could underlie a potentially large number of cases of preventable blindness. Using risk factors for glaucoma to provide guidelines for targeting at-risk groups, to improve early glaucoma detection and treatment are currently the most powerful tools for preventing blindness and low vision in this predominantly asymptomatic disease in its early stages.
